# Transformer and graph variational autoencoder to identify microenvironments: A deep learning protocol for spatial transcriptomics

**DOI:** 10.1016/j.xpro.2025.104206

**Published:** 2025-11-27

**Authors:** Karla Paniagua, Yufei Huang, Shou-jiang Gao, Yidong Chen, Yu-Fang Jin, Mario Flores

**Affiliations:** 1 Department of Electrical and Computer Engineering, KLESSE School of Engineering and Integrated Design, University of Texas at San Antonio, San Antonio, TX 78249, USA; 2 Cancer Virology Program, UPMC Hillman Cancer Center, University of Pittsburgh School of Medicine, Pittsburgh, PA, USA; 3 Department of Medicine, University of Pittsburgh School of Medicine, Pittsburgh, PA, USA; 4 Department of Electrical and Computer Engineering, Swanson School of Engineering, University of Pittsburgh, Pittsburgh, PA, USA; 5 Department of Microbiology and Molecular Genetics, University of Pittsburgh School of Medicine, Pittsburgh, PA, USA; 6 Greehey Children Cancer Research Institute, The University of Texas Health Science Center at San Antonio, San Antonio, TX 78229, USA; 7 Department of Population Health Science, The University of Texas Health Science Center at San Antonio, San Antonio, TX 78229, USA

**Keywords:** Bioinformatics, Sequence analysis, Single Cell, Cancer, Genomics, Gene Expression

## Abstract

We present transformer and graph variational autoencoder to identify microenvironments (TG-ME), a computational framework that integrates transformer and graph variational autoencoders to dissect spatial niches using spatial transcriptomics and morphological images. This protocol outlines data normalization, spatial transcriptomics integration, morphological feature extraction, and niche profiling. Using deep learning, TG-ME enables robust niche clustering applicable to healthy, tumor, and infected tissues.

For complete details on the use and execution of this protocol, please refer to Paniagua et al.^1^

## Highlights

• Workflow to build spatial objects linking stitched images with transcriptomics data
• Steps to apply transformer and GraphVAE for spatial and contextual tissue modeling
• Guide to merge gene expression and histology data into one spatial framework
• Instructions for clustering and deconvolution to map tumor niches at single-cell level

## Abstract (editor highlights)

Publisher’s note: Undertaking any experimental protocol requires adherence to local institutional guidelines for laboratory safety and ethics.

## Graphical abstract

**Figure:**
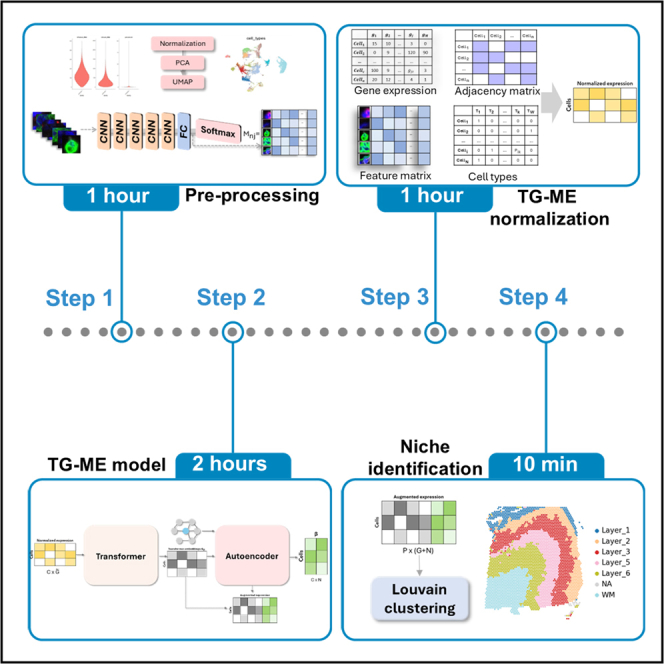

## Before you begin

Recent advances in Spatial transcriptomics (ST) technologies combine imaging and sequencing methods to obtain gene expression data, spatial cell or spot locations, and morphology images.^2^^,^^3^ These advancements provide insights into the function and heterogeneity of spatial organization structures.^4^ However, identifying microenvironments from this multimodal, high dimensional data becomes a challenging task.^5^ There is a need for innovative tools that can analyze and interpret this type of dataset.

Deep learning (DL) methods have been developed for several biological tasks and demonstrated to be well-suited for handling the challenges presented by ST data.^1^^,^^6^^,^^7^^,^^8^^,^^9^^,^^10^^,^^11^^,^^12^^,^^13^^,^^14^^,^^15^ Some existing methods have focused on dissecting spatial niches in ST data using different DL approaches.^12^^,^^16^^,^^17^^,^^18^ However, common challenges remain, including the complexity of the multimodal and high dimensional ST datasets, which makes integrating data types difficult.

To overcome these challenges, this protocol introduces Transformer and a Graph Variational Autoencoder to identify microenvironments (TG-ME), a DL framework for dissecting spatial niches in high-resolution ST data. Building on our previous study,^1^ this protocol provides a comprehensive guide for integrating the different modalities from ST data, extracting morphological features, and identifying spatial niches in healthy, infected or diseased samples. The following sections provide step-by-step instructions for identifying the microenvironment and dissecting the niches in a high-resolution spatial transcriptomics sample from.^2^ Prior knowledge of Python is required.

### Innovation

TG-ME introduces an integrated deep learning framework that combines Transformer models with a Graph Variational Autoencoder (GraphVAE) to dissect spatial niches in high-resolution spatial transcriptomics datasets. Unlike prior approaches such as SpaGCN, DeepST, or adaptive graph autoencoders that focus on either spatial or transcriptomic modalities, TG-ME simultaneously incorporates gene expression, morphological features, spatial coordinates, and cell type annotations. This multimodal integration enables more accurate niche detection across both single-cell and spot-level datasets.

Key innovations of TG-ME include multimodal integration, as it jointly processes gene expression matrices, morphology-derived features, and spatial neighborhood information within a single framework. It offers cross-platform compatibility: while optimized for Nanostring CosMX datasets, TG-ME can also be applied to 10× Visium and Xenium data, making it broadly adaptable to different resolutions and platforms. The framework leverages transformer-based gene representation, where self-attention mechanisms capture gene-gene interactions within each cell, enhancing interpretability and robustness. In addition, the Graph Variational Autoencoder encodes cell-cell spatial relationships, enabling biologically meaningful niche clustering. Finally, TG-ME provides a reproducible computational workflow by offering standardized preprocessing (normalization, cropping, feature extraction) and detailed training instructions, reducing variability and enhancing reproducibility.

Together, these features position TGME as an advancement over existing spatial transcriptomics analysis tools, offering researchers a practical and extensible method for mapping microenvironments in healthy, infected, or diseased tissues.

### Environment installation

**Timing: 30 min to 1 h**

Since this protocol is based on a Deep Learning framework composed of a Transformer and a Graph Variational Autoencoder for high-resolution spatial transcriptomics data, it is computationally intensive. For small test datasets, a local computer with a quad-core CPU, 16 GB of RAM, and a mid-range GPU (e.g., NVIDIA GTX 1660) may suffice for limited inference. However, for realistic datasets or full-resolution analysis, a high-performance computer or server is strongly recommended. In that case, a system with an 8–12 core CPU, 32–64 GB of RAM, a high-end GPU with at least 24 GB of VRAM (e.g., NVIDIA RTX 4090 or A6000), and SSD storage of 1 TB or more will provide the necessary resources to efficiently run the protocol. Utilizing a high-performance setup will significantly reduce computation time and memory issues, especially when training or performing inference on large-scale spatial data.

1. Install the environment:
  a. Download the corresponding “.yaml” file from https://github.com/Karladanielap/TG-ME/tree/main/Installation.
    ***Note:*** If you have Windows or Linux systems select “TGME2-Windows.yaml”. If you have a Mac system select “TGME2-MAC.yaml”.
  b. Open the Powershell Prompt in Windows, terminal in Mac, and go to the folder where you saved the file.
    ***Note:*** If you are working on Windows, make sure you have rustic installed:
    > rustc --version
    if not, you can install through https://rustup.rs/.
  c. To install the environment, type the following lines:
    ***Note:*** Use Anaconda Powershell Prompt on Windows or the Terminal on Mac.
    >conda update -n base conda # for Windows
    >conda install conda=24.9.2 # for Linux
    >conda update -n base anaconda-navigator
    >pip install requests --upgrade
    >conda env create -f TGME2.yaml --name TGME
    >conda activate TGME
    >conda install anaconda::ipykernel
    > python -m ipykernel install --user --name TGME
    --display-name "TGME"
    > jupyter notebook
    ***Note:*** These steps might take some time installing the environment depending on your equipment.
    ***Note:*** Make sure you have installed the scikit-network package version 0.20.
2. Install PyTorch.
  a. To install PyTorch go to https://pytorch.org/get-started/locally/.

***Note:*** Confirm that the suggested PyTorch configuration matches your operating system and compute platform.

***Note:*** If you install through pip in Jupyter Notebook PyTorch will suggest installing using “pip3” do it with “pip” instead.

3. Install PyTorch dependencies torch-sparse, torch-scatter and torch-geometric.

> pip install pyg_lib torch_scatter torch_sparse torch_cluster torch_spline_conv -f https://data.pyg.org/whl/torch-${TORCH}+${CUDA/CPU}.html

***Note:*** Refer to https://data.pyg.org/whl/ to select the correct version depending on your torch version and CUDA availability and version.

**CRITICAL:** The previous steps are critical for the success of the protocol.

### Download the data and model code

**Timing: 10 min**

4. Download the raw data (key resources table).

***Note:*** If you use the lung data, this protocol was designed with Lung 5 Rep 1. Download for “Lung 5-1” the “Data Files’ and the “Raw Morphological Images”.

***Note:*** If you use the pancreas dataset, download the “Basic_Data_Files”.

5. Download the code files (key resources table).

***Note:*** We suggest you clone the repository. Make sure you include the files in the same folder that you are going to be working on.

***Note:*** The cell typing can be replaced by the clustering annotation if you do not have this information.

***Note:*** We recommend saving the code in your working folder.

***Note:*** In this protocol, we provide an example using high-resolution spatial transcriptomics data generated with Nanostring’s CosMX technology; however, the workflow is not limited to this platform and can also be applied to other technologies, such as 10× Visium and Xenium.

## Key resources table

**Table:** 

REAGENT or RESOURCE	SOURCE	IDENTIFIER
**Deposited data**		

Nanostring CosMX Non-Small Cell Lung Cancer	Shanshan et al.^2^	https://nanostring.com/products/cosmx-spatial-molecular-imager/ffpe-dataset/nsclc-ffpe-dataset/
Nanostring CosMX Pancreas	Nanostring	https://nanostring.com/products/cosmx-spatial-molecular-imager/ffpe-dataset/cosmx-smi-human-pancreas-ffpe-dataset/
Cell type annotations		https://github.com/Karladanielap/TG-ME/tree/main/Cell-Types

**Software and algorithms**		

Python package Scanpy 1.10.1	Wolf, Angerer, and Theis^19^	https://genomebiology.biomedcentral.com/articles/10.1186/s13059-017-1382-0
Python package Pytorch 1.13.1+cu116	Paszke et al.^20^	https://proceedings.neurips.cc/paper/2019/hash/bdbca288fee7f92f2bfa9f7012727740-Abstract.h
Python package Pandas 2.2.2	The pandas development team	https://zenodo.org/records/10957263
Python package Numpy 1.26.0	Harris et al.^21^	https://doi.org/10.1038/s41586-020-2649-2
Python package anndata 0.10.7	Virshup et al.^22^	https://www.biorxiv.org/content/10.1101/2021.12.16.473007v1
TG-ME code	Paniagua et al.^1^	https://github.com/Karladanielap/TG-ME/

## Step-by-step method details

**Figure 1:**
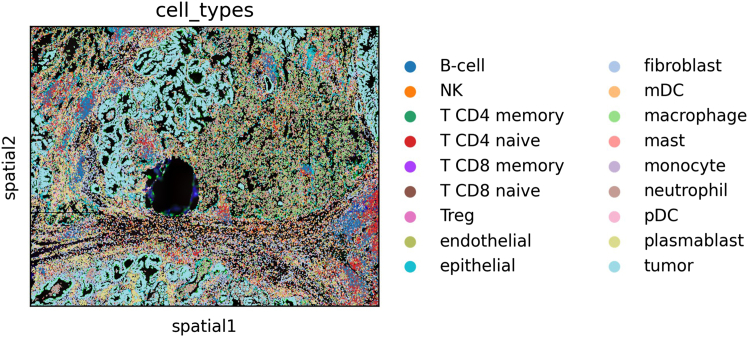
Spatial object plotting the cell types

This section provides detailed step-by-step instructions for the protocol, including data pre-processing, normalization, model training, and niche identification, as illustrated in Figure 1.

### Load the required packages

**Timing: 3 min**

In this step, we import all necessary Python libraries and TG-ME functions required to execute the protocol. This ensures that all dependencies for spatial data handling, preprocessing, visualization, and deep learning are available before beginning the analysis.

1. Load all Python libraries and TG-ME functions necessary to execute the protocol.

***Note:*** The functions for TG-ME are available in the GitHub repository.

***Note:*** Import warnings are suppressed for cleaner output. Make sure all packages are installed in the TG-ME environment.

>from stitch2d import StructuredMosaic

>import pandas as pd

>import numpy as np

>import scanpy as sc

>import anndata

>import warnings

>warnings.filterwarnings('ignore')

>from anndata import AnnData

>import squidpy as sq

>from numpy.random import default_rng

>import matplotlib.pyplot as plt

>import matplotlib.image as mpimg

>import os

>from pathlib import Path

>from scipy.sparse import issparse, isspmatrix_csr, >csr_matrix, spmatrix

>from sklearn.metrics import pairwise_distances

>from sklearn.decomposition import PCA

>from sklearn.metrics import adjusted_rand_score

>from sklearn.metrics import report

>from tqdm import tqdm

>import random

>from PIL import Image

>import torch

>import torch.nn

>import torchvision.models as models

>from torch.autograd import Variable

>import torchvision.transforms as transforms

>import pickle

>import gzip

>from sklearn.preprocessing import LabelEncoder

# TG-ME functions

>from stitching_coords import modify_coordinates_raster

>from stitching_coords import modify_coordinates_snake

>from spatial_object import spatial_object

>from preprocessing import preprocessing

>from preprocessing import image_crop

from preprocessing import image_feature

from TGME_norm import run

from TGME_norm import celltypes_encoding

from TGME_norm import dim_red_augmented

### Object stitching (preprocessing)

***Optional:*** Some spatial transcriptomics datasets are acquired as multiple fields of view (FOVs) rather than a single whole-tissue image. Before analysis, these FOVs must be reconstructed into a single stitched image and a corresponding coordinate system to ensure that morphology, cell coordinates, and gene expression are correctly aligned. This preprocessing step does not affect gene expression or cell annotations but provides the necessary spatial reference framework for downstream analyses. We created two functions under “stitching_coords.py” to support this section.

***Note:*** Examples for raster and snake schemes are provided in the Supplementary Material (Figures S1–S3).

2. Use the function modify_coordinates_raster or modify_coordinates_snake to adapt the coordinates to the stitched image.

### Spatial object

**Timing: 20 min**

In this step, we create a *spatial object* that integrates the stitched morphology image, the corresponding cell coordinates, and the gene expression data for a sample. This object serves as the foundation for all subsequent analyses.

3. Load the stitched metadata.

>df=pd.read_csv('/path/to/save/stitched_metadata.csv')

***Note:*** If you are using the Lung data, use the idx column as index: index_col='idx':

>df=pd.read_csv('/path/Lung5_stitched_metadata.csv',index_col='idx')

4. Load the gene expression matrix.
  >exp=pd.read_csv('/path/Lung5_stitched_metadata.csv',index_col='idx',index_col='Unnamed: 0')
  ***Note:*** If you are using the Pancreas sample we need to remove the FOVs from the second sample. We also need to set the index of the cells.
  ***Note:*** If you are using the Lung data, use the idx column as index: index_col='idx': df=pd.read_csv('/path/to/save/Lung5_stitched_metadata.cs v',index_col='idx')
  a. Remove FOVs from sample 2.
    >exp=exp[exp['fov']<60]
  b. Set index using cell ID and FOV.
    >exp['index']=exp['fov'].astype(str)+'-'+exp['cell_ID'].astype(str)
    >exp.set_index('index',inplace=True)
  c. Remove columns.
    >exp.drop(columns=['fov','cell_ID'],inplace=True)
5. Define paths to save data.

>data_path = "/path/to/data/" #### to your path

>data_name = 'Lung' #### project name

>save_path = "/path/to/save" #### save path

6. Call the spatial_object function available in the *spatial_object.py* file to create the spatial object. This function will take the following components as input:
  a. df, which is going to be the metadata (with the stitched coordinates).
  b. exp, which is the gene expression matrix.
  c. img, which is the path to the stitched image.
  d. name, which is the name of the tissue (e.g., ‘Lung’).

>adata= spatial_object(df,exp,img,name)

7. Optional: Add the cell typing if available, the width and height of the cells, and any other metadata information that you want to include in the analysis.
  a. Download the cell types from: https://github.com/Karladanielap/TG-ME/tree/main/Cell-Types.
    ***Note:*** If the cell typing is not available for your data, or by some reason you cannot compute it, you can replace it with the clustering information. If that is the case, you will not need to load anything on this point.
  b. Make sure the index of the cell typing data frame and your object are in the same format.
    >celltypes=pd.read_csv('/path/to/cell_type-Lung.csv')
    >celltypes['cell_ID']=celltypes['cell_ID'].str.replace('c_1_','')
    >celltypes['cell_ID']=celltypes['cell_ID'].str.replace('_','-')
    >celltypes.set_index('cell_ID',inplace=True)
  c. Add the cell types, the width and the height of the cells.
    >adata.obs['cell_types']=celltypes['cell_types']
    >adata.obs['Width']=df['Width']
    >adata.obs['Height']=df['Height']
8. Save spatial object.

>adata.write('Lung5_data.h5ad')

### Preprocessing

**Timing: 1 h**

This step involves data normalization, dimensionality reduction, clustering, and generating the UMAP. It is followed by cropping individual cell images and extracting their morphological features for subsequent analysis.

**Figure 2:**
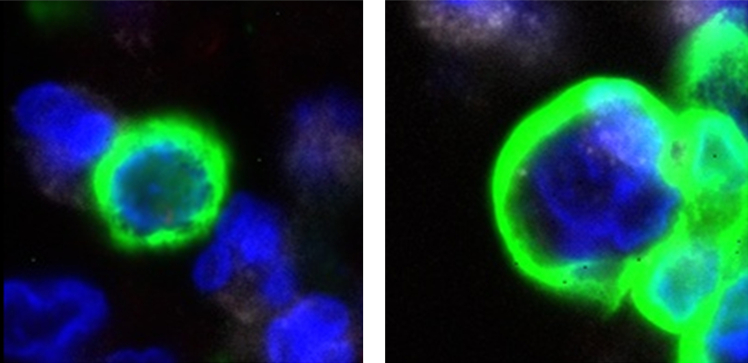
Examples of image cropping for 2 different cells

9. Data pre-processing.
  ***Note:*** For this step, use the TG-ME defined function, called preprocessing available, in the *preprocessing.py* file. The input should be the spatial object.
  ***Note:*** This function will automatically normalize and scale the data, compute the principal component analysis (PCA), identify the nearest neighbors, compute the uniform manifold approximation and projection (UMAP), and compute the clustering using Leiden.
  >adata= preprocessing(adata)
  a. Visualize the cell types in their spatial location.
    >sq.pl.spatial_scatter(adata, color="cell_types",size=30, dpi=300, save='Lung5-spatial-cell-types.png')
10. Image cropping.
  ***Note:*** The second step of the preprocessing includes cropping the images of the individual cells.
  a. Determine and/or create the path where the cell images will be saved.
    >data_name = 'Lung5' #### project name
    >save_path = "/path/to/images/" #### save path
    >save_path_image_crop = Path(os.path.join(save_path, 'Image_crop', f'{data_name}'))
    >save_path_image_crop.mkdir(parents=True, exist_ok=True)
  b. Call the TG-ME function, called image_crop, defined in the *preprocessing.py* file. The input will be:
    i. adata, which is the spatial object.
    ii. save_path, which is the location where you are going to save the cropped images.
    iii. library_id, the name of the tissue that you set when creating the spatial object.
    iv. crop_size, which is a string used when saving the image.
    v. targeted_size, that is targeted image size.
    vi. verbose, if we want to track progress.
      >adata = image_crop(adata, save_path, library_id=None,crop_size='fit', target_size=224, verbose=False, )
      ***Note:*** This step takes about 60 min to complete but may vary depending on your system. It also depends on the size of the image. Larger images may take longer.
      ***Note:*** The function will automatically save the location of the images in the spatial object.
      ***Note:*** From the previous step, we obtain a set of cropped images, each corresponding to a single cell (Figure 2). Each image is centered on the cell’s coordinates and standardized to the same size, based on the largest cell. This ensures that every cell is represented consistently as an individual image rather than as part of the whole tissue.
11. Extract morphological features.
  ***Note:*** The next step is to extract morphological features from these per-cell images so they can be incorporated into the analysis.
  a. For this, use the TG-ME function, image_feature, defined in the *preprocessing.py* file. The input should contain two elements:
    i. adata, which is our spatial object.
    ii. pca_components, which is the number of PCA that we want to obtain from the image features.
      ***Note:*** The function will automatically save the feature matrix in the spatial object.
      >adata = image_feature(adata, pca_components).extract_image_feat()
      ***Note:*** This step takes about 3 h without GPU. And the function will automatically check if there is a GPU available and use it if that is the case.

### TG-ME normalization and graph creation

**Timing: 1 h**

In this section, we implement the TG-ME normalization method which enables the integration of multimodal information from spatial transcriptomics data, including gene expression, morphological images, spatial coordinates, and cell type annotations. We also construct a ball tree graph and derive an adjacency matrix from it to identify neighboring cells.

12. Define that we will use TG-ME functions using the GPU, if available, if not set to False.

>TGME = run(use_gpu=True)

13. Encode the cell types.
  a. For this, we will call the TG-ME function, celltypes_encoding, available on the *TGME_norm.py* file. The input to this function includes:
    i. adata which is our spatial object,
    ii. cell_types which is a string with the name of the column that contains the cell types.

>adata = celltypes_encoding(adata,cell_types)

14. Store the name of the cell types in a separate function.

>ct=adata.obs.cell_types.unique()

15. Perform the TG-ME normalization.
  ***Note:*** In this step we use a modified function obtained from.^16^ The function is available in the *augment.py* file.
  ***Note:*** This function normalizes the data as a function of spatial location, morphology features, gene expression and cell type.
  The inputs include.
  a. ct which is a list of the unique cell types.
  b. adata which is our spatial object.
  c. adjacent_weight which is the weight that we want to give to adjacent neighboring cells, and the number of neighbors we want to select for the analysis. Default values work for most of the cases.
    >adata = TGME._get_augment(ct,adata, adjacent_weight = 0.8, neighbour_k = 12)
16. Compute the graph using the function get_graph available in the augment.py file. As input include:
  a. adata.obsm["spatial"] which is the location of the spatial information is stored in our spatial object.
  b. distType which is the type of graph we want to use.
  c. k which is the number of neighbors.
    ***Note:*** We recommend using the same number of neighbors that in the previous function.
    >graph_dict = TGME._get_graph(adata.obsm["spatial"], distType="BallTree", k=12)
  d. Save the graph.
    >with gzip.open("graph-Pancreas.pkl.gz", "wb") as compressed_file:
    >pickle.dump(graph_dict, compressed_file)

### Identification of tissue microenvironments using TG-ME

**Timing: 2 h**

In this section, we split the data into training, testing, and validation sets. We then train the TG-ME model and use the resulting pre-trained model to extract the final embeddings. These embeddings are used to identify the microenvironment through clustering.

***Note:*** Use the objects previously generated with the pipeline above.

17. Start by encoding the cell types, as they are originally in text format and need to be converted to numerical labels for use in the analysis.

***Note:*** If the cell type is missing, and you are using your cluster labels, we still recommend doing this step.

>label_encoder = LabelEncoder()

>y_label= label_encoder.fit_transform(adata.obs['cell_types'])

18. Call the TG-ME function *dim_red_augmented()* available in the *TGME_norm.py* file to extract the top 50 principal components (PCs) from the TG-ME normalized data to reduce computational complexity.

***Note:*** This dimensionality reduction helps decrease training time and improves processing efficiency. The 50 PCs are saved in the variable concat_X. You can select the desired number of PCs.

>x=dim_red_augmented(adata,50)

19. Prepare the data for training the model.
20. Load the TG-ME function splitdata() available in the *TGME.py* file. The function uses as input:
  a. Num_classes which is the number of classes (cell types or cluster labels) available on your dataset.
  b. test_size the desired size (percentage) of your testing set.
  c. val_size the desired size of your validation set.
  d. x the dimensionality reduction of your TG-ME normalized data.
  e. y_label the encoded cell types.
  f. graph_dict the graph.

***Note:*** This function will help us split the data into training and testing sets, and define data loaders that will convert our variables into tensors. Converting the data into tensor format is essential when using PyTorch, as tensors are the core data structure that PyTorch models operate on. This format enables efficient computation on GPUs and seamless integration with the PyTorch training pipeline.

***Note:*** You can define the training, testing and validation percentages according to your needs. In this protocol we selected one of the standard 80% for training, 10% testing and 10% for validation.

>X_train, y_train, X_val, y_val, X_test, y_test,train_graph,val_graph,test_graph= splitdata(num_classes,test_size,val_size,x,y_label,graph_dict)

21. Next, call the function CreateDataLoaders() available in the *TGME.py* file to load the data. The input will be:
  a. X_train, X_test, X_val: the training, testing and validation data.
  b. Y_train, y_test, y_val: the training, testing and validation labels.
  c. train_graph,val_graph,test_graph: the training, testing and validation graphs.
  d. batch_size: the size of the batch to use while training.

>train_loader,val_loader,test_loader=CreateDataLoaders(X_train, y_train, X_val, y_val, X_test, y_test,batch_size)

22. Create the graph loaders. For this call the GraphLoaders() function available in the *TGME.py.* The input will be:
  a. train_graph,val_graph,test_graph: the training, testing and validation graphs.
  b. batch_size: the size of the batch to use while training.

>dataloader_trainG=GraphLoader(train_graph, 128)

>dataloader_testG=GraphLoader(test_graph, 128)

>dataloader_valG=GraphLoader(val_graph, 128)

***Note:*** The graph loader will be useful for loading the data into our Graph Variational Autoencoder model.

23. Train the model.
24. Start by defining the model parameters, which include:
  a. Batch_size: The size of the batch you are going to be using during training. Here you need to define the same value you used for creating your data and graph loaders.
  b. n_head: the number of heads to use in the transformer model.
  c. n_gene: the number of genes or PCs in your training data.
  d. num_classes: The number of classes in your dataset.
  e. d_ff: the feed forward dimension.
  f. dropout_rate: the dropout rate (if any) that you want to use during training in the transformer.
  g. Mode: a flag to train the transformer on the gene expression.
  h. input_dim: is the same as the n_gene but for the graph variational autoencoder.
  i. conv_hidden: the size of your hidden layers.
  j. p_drop: dropout rate in the graph variational autoencoder.
  k. dec_cluster: the number of classes.
  l. Activate: the activation function.

**CRITICAL:** These parameters may vary according to your dataset. We recommend to add dropout as it helps the model to better learn the low dimensional representations and it can prevent overfitting.

# Define TG-ME parameters

>batch_size = 128

>n_head = 3

>n_gene = 50

>num_classes=16

>d_ff = 1024

>dropout_rate = 0.7

>mode = 0

>input_dim=50

>conv_hidden=[32,16]

>p_drop=0.1

>dec_cluster_n=num_classes

>activate="relu"

num_epochs = 500

verbose=True

verbose_interval=10

lr=0.001

weight_decay=0.7

25. Create the model by calling the TG-ME function called TGME available in the *TGME.py*.

>model = TGME(batch_size, n_head, n_gene,num_classes, d_ff, dropout_rate, mode, input_dim, conv_hidden, p_drop, dec_cluster_n,activate)

26. Define the number of epochs.

***Note:*** The training function is designed with early stopping, so the model might not run all the epochs if it detects it stopped learning.

***Note:*** Set verbose to True if you want progress information while training, and the verbose_interval to optain it every defined number of epochs.

**CRITICAL:** Set your learning rate (*lr*), this will vary depending to your data. Larger *lr* values will train the model faster. Define the weight_decay if needed.

>num_epochs = 500

>verbose=True

>verbose_interval=10

>lr=0.001

>weight_decay=0.7

27. Train the model. For this, call theTG-ME function train_TGME() from *TGME.py.* The inputs will include the model, the parameters previously defined, the data loaders, and the graph loaders and the path where you want to save the trained model (path_save).

>model=train_TGME(model,lr,weight_decay,num_epochs,verbose,verbose_interval,train_loader,val_loader,dataloader_trainG,dataloader_valG,path_save)

28. Obtain the final embeddings.

Call the TG-ME data loader functions AllDataLoader() and graph loader GraphLoader() from the *TGME.py* file to load all the data and extract the features.

29. Create the data loader.

>dataloader_G=GraphLoader(graph_dict, 128)

>X=np.array(x)

>X_dataloader=AllDataLoader(X, y_label, 128)

30. Load the saved model.

>model.load_state_dict(torch.load(path_saved_model))

***Note:*** You should see the legend: *<All keys matched successfully>* after loading the model, meaning that the model was successfully loaded.

31. Set the model as pretrained_model.

>pretrained_model=model

32. Extract the features by calling the TG-ME function extract_embeddings() from the *TGME.py* file. As input use the pretrained model and the data and graph loaders.

>embeddings=extract_embeddings(pretrained_model, X_dataloader, dataloader_G)

***Note:*** The output will be a data frame with the final embeddings.

33. TME identification.
34. Cluster the embeddings to identify niches. For this, input the identified embeddings, spatial object (adata), the number of neighbors to use (n_neighbors), and the clustering resolution (resolution).

***Note:*** These parameters should be modified according to your data. Smaller resolution values will give less clusters. Usually, you would like to have less clusters than cell types, as several cell types can be grouped in niches. We recommend evaluating if your resolution is good by visualizing the spatial niches, and evaluating the cell type percentages per niche.

>adata= TGME_clustering(embeddings,adata, n_neighbors,resolution)

***Note:*** The output will be the spatial object with the clustering of the niches, saved in adata.obs[‘TG-ME_refine_domain’].

35. Finally, visualize the refined clusters.

>sc.pl.spatial(adata, color=['TG-ME_refine_domain'], spot_size=80,img_key=None,palette='tab20')

***Note:*** Niches (clusters) can be further annotated based on their cell type composition, or any other desired method. Downstream analysis can be performed on the identified niches.

## Expected outcomes

The TG-ME (Transformer-Graph Multimodal Embedding) protocol is designed to dissect tumoral niches within the tumor microenvironment by integrating spatial transcriptomics data, cell morphology, and spatial localization through a deep learning framework. When the protocol is applied, researchers can expect to generate niche annotations across tissue samples. This includes the identification of spatially organized cellular subpopulations and detailed maps of tissue microarchitecture, offering a comprehensive view of tumor heterogeneity.

**Figure 3:**
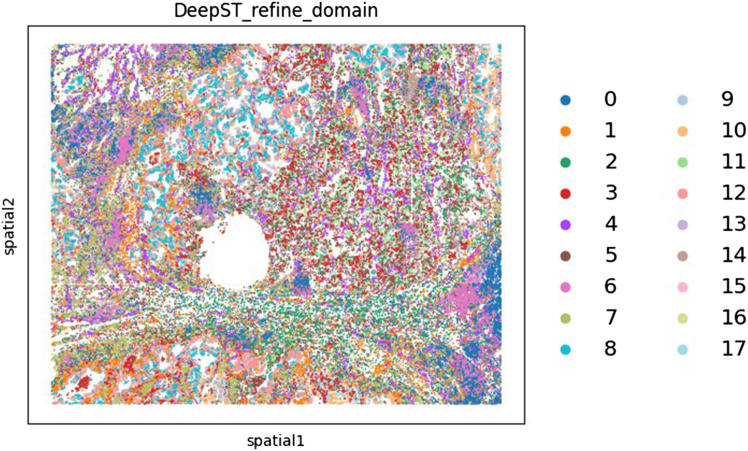
Niches obtained from the NSCLC dataset with a resolution of 0.81

**Figure 4:**
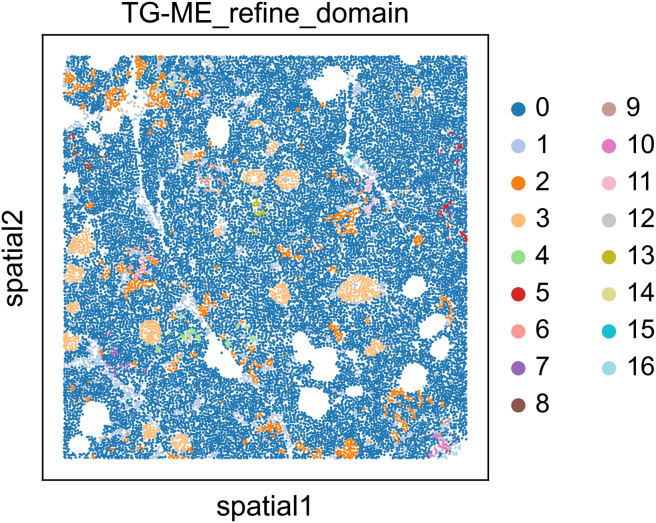
Niches identified on the Pancreas dataset with a resolution of 0.1

Specifically, the protocol produces several key data outputs. These include normalized and integrated gene expression matrices that incorporate gene expression, morphology features, spatial proximity, and cell type information. The Transformer model outputs attention maps that capture gene-gene interactions within individual cells, while the GraphVAE module produces latent embeddings that encode spatial relationships between cells. Together, these models enable the detection of distinct tumor and stromal niches via unsupervised clustering (Figures 3 and 4).

Researchers can employ performance metrics such as precision, recall, F1-score, adjusted Rand index (ARI), and normalized mutual information (NMI), to quantify the quality of niche classification. Upon successful execution of the TG-ME pipeline, researchers will be able to characterize the molecular features of each niche through downstream analysis.

Overall, the anticipated outcome of the TG-ME protocol is a detailed, biologically interpretable map of tumor microenvironments at the single-cell level, when high resolution data is available, and spot level. This enables researchers to explore spatial organization, uncover cell-cell interactions, and generate hypotheses regarding tumor progression, immune evasion, and potential therapeutic targets.

## Limitations

The TG-ME protocol has several limitations that should be considered when applying it to spatial transcriptomics datasets. In severe tumor stages, cellular heterogeneity becomes highly dysregulated, with a large proportion of cancer cells, which reduces the model’s ability to distinguish cell types and results in lower precision, recall, F1-score, ARI, and NMI. Although niche-level or pathologist annotations are typically unavailable, these metrics can still be estimated during model training using existing cell type or clustering annotations to assess whether the model is learning meaningful latent representations. Another limitation arises when cell typing information is absent. The model requires cell typing to run, and while clustering-derived labels or reference-based annotations can be substituted, these strategies often reduce accuracy compared to true cell types. The protocol also depends on multimodal data, requiring the integration of gene expression, morphology, spatial coordinates, and cell type information. If any of these modalities are missing, the model may still operate but performance will decrease substantially, as shown in ablation studies.

Platform compatibility also influences performance. TG-ME was tested on 10× Visium and NanoString CosMX datasets; while Visium does not provide single-cell resolution and lacks direct cell type annotations, the model can still be applied using spot-level or clustering-derived labels. However, batch effects introduced by sample preparation, sequencing depth, or platform-specific biases can alter results, though these can be partially mitigated with existing batch correction tools such as ComBat, Harmony, or Seurat integration. Another limitation is the computational demand of the framework. Large datasets with hundreds of thousands of cells require high-performance computing resources, particularly GPUs with at least 16–24 GB of memory. Running on high-performance clusters mitigates these issues, but usability is constrained in low-resource environments.

Reproducibility and generalization also present challenges. Consistent adherence to the recommended preprocessing pipeline is essential, as deviations (e.g., alternative normalization methods) may lead to divergent results. Similarly, generalization may be limited when applying TG-ME to datasets with very different resolutions, platforms, or biological contexts. Despite these challenges, the protocol is broadly compatible with any spatial transcriptomics dataset at either spot-level or single-cell resolution, as long as spatial coordinates and morphology or H&E images are available. Annotated cell types enhance performance but are not strictly required, since clustering-derived labels can be used instead. Together, these considerations highlight the need to balance biological, technical, and computational constraints when applying TG-ME to complex datasets.

## Troubleshooting

### Problem 1

During the installation process, after running.

>conda update -n base conda # for Windows

>conda install conda=24.9.2 # for Linux

>conda update -n base anaconda-navigator

If you see the error ImportError: cannot import name ‘JSONDecodeError’ from ‘requests.exceptions’.

### Potential solution

Run the following code.

>pip install requests --upgrade

Then repeat the installation process.

### Problem 2

For any problems related to the dependencies.

### Potential solution

Go back to the “TGME.yaml” file, look for the required version, and reinstall the package making sure you are utilizing the correct version.

>pip install pandas==1.5.0

### Problem 3

For problems related to incompatibility between PyTorch and its dependencies including torch-sparse, torch-geometric, pyg.

### Potential solution

First verify the installed PyTorch version.

>import torch

>print(torch.__version__)

After identifying the installed version, re-install torch-sparse, torch-geometric, pyg. For this, you can go to https://data.pyg.org/whl/find the folder with the version of PyTorch that you have installed, and select CUDA if your system has a GPU or CPU otherwise. After identifying the package, you can installed it using the following command, making sure you replace ${TORCH} for the installed PyTorch version, and ${CUDA/CPU} depending on GPU availability on your system.

> pip install pyg_lib torch_scatter torch_sparse torch_cluster torch_spline_conv -f https://data.pyg.org/whl/torch-${TORCH}+${CUDA/CPU}.html

### Problem 4

For problems installing PyTorch, where you get an error message similar to “ERROR: Could not find a version that satisfies the requirement torch (from versions: none)”.

### Potential solution

This error is related to incompatibility across python version and new PyTorch versions. PyTorch versions > 12.6 are not compatible with Python version ≤ 3.8. You can install PyTorch versions ≤ 12.4 or upgrade python.

### Problem 5

Training fails, runs out of memory, or is extremely slow.

### Potential solution

Ensure sufficient GPU memory (≥16–24 GB recommended for large datasets). For very large datasets (>300,000 cells), consider running on a High-Performance Computing (HPC) cluster. Batch size can be reduced to fit GPU memory, though this may increase training time.

### Problem 6

Model predictions appear unstable or accuracy is low.

### Potential solution

Verify that the preprocessing pipeline has been followed precisely, including normalization and feature selection steps. Check that the correct cell type annotations or clustering-derived labels are provided.

### Problem 7

Morphology images or spatial coordinates fail to integrate with TG-ME.

### Potential solution

Ensure that all images are correctly linked to the cell identifiers in the spatial object and that image file paths are accessible. Confirm that spatial coordinates match the cell order in the data matrix.

### Other problems

• **Reloading trained models or pickled graphs:** There should be no issues with reloading the trained TG-ME model or pickled graph files, and we have not encountered problems in practice.
• **Compatibility with downstream tools:** All presented tools are compatible with downstream analyses. However, some Python packages may have version conflicts that could affect reproducibility or functionality. To prevent this, we provide .yaml environment files at the beginning of the protocol to create the environment with package versions tested for compatibility.
• **Starting from an existing AnnData object:** This is possible, but the AnnData must contain all resources required by TG-ME, including cell spatial coordinates, morphology images, and associated metadata. These components are essential for TG-ME normalization and integration. As long as the AnnData object includes this information (whether created de novo or converted from other formats such as Seurat), compatibility is maintained.

## Resource availability

### Lead contact

Further information and requests for resources and reagents should be directed to and will be fulfilled by the lead contact, Mario Flores (mario.flores@utsa.edu).

### Technical contact

Technical questions on executing this protocol should be directed to and will be answered by the technical contact, Karla Paniagua (karla.paniaguarivera@my.utsa.edu).

### Materials availability

This study did not generate new unique reagents.

### Data and code availability

• Original data for analysis in the paper are available at https://doi.org/10.1038/s41587-022-01483-z and https://nanostring.com/products/cosmx-spatial-molecular-imager/ffpe-dataset/cosmx-smi-human-pancreas-ffpe-dataset/.
• The code analyzed during this study is available at https://github.com/Karladanielap/TG-ME/.
• Zenodo DOI: https://doi.org/10.5281/zenodo.17229338.

## Acknowledgments

This article’s publication costs were supported partially by the National Institutes of Health (3R01CA124332-13A1S1 to M.F.), the National Science Foundation (2051113 to Y.-F.J.), the National Institutes of Health (P30 CA054174 to Y.C.), Cancer Prevention and Research Institute of Texas Core (RP220662 to Y.C.), and the National Institutes of Health (R01 CA284554 to S.-j.G.).

## Author contributions

K.P. was responsible for coding, data acquisition, interpretation, and drafting the protocol. M.F. was responsible for data acquisition, interpretation, and validation and drafted and critically revised the protocol. Y.-F.J. and M.F. contributed to the conceptualization and analysis and also drafted and critically revised the manuscript. Y.C., S.-j.G., and Y.H. provided resources, contributed to writing, and approved the final version of the manuscript. All authors have read and approved the final manuscript.

## Declaration of interests

The authors declare no competing interests.
