# Supplementary material for: Transformer and graph variational autoencoder to identify microenvironments: A deep learning protocol for spatial transcriptomics
Source: STAR Protoc. 2025 Nov 27;6(4):104206. doi: 10.1016/j.xpro.2025.104206 (PMC12702057; doi:10.1016/j.xpro.2025.104206)
Supplement: Document S1. Figures S1–S3 [file mmc1.pdf]

Supplemental materials

Object Stitching

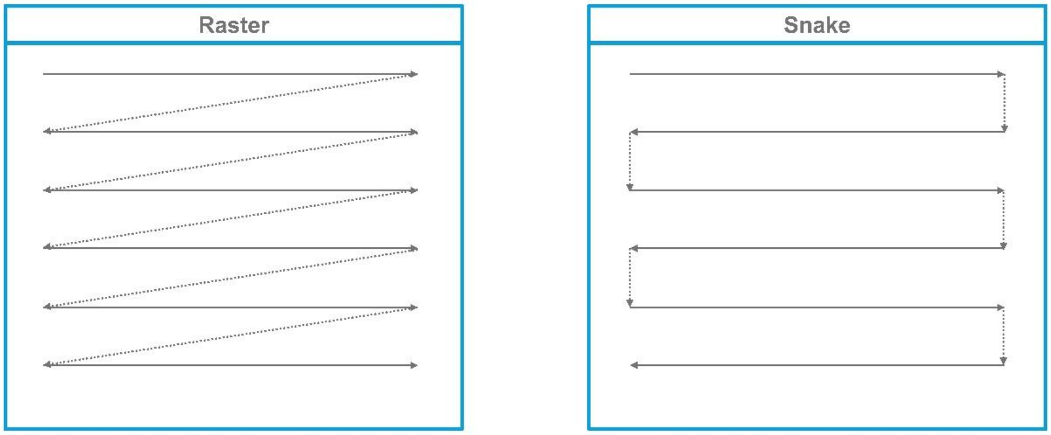

Figure S1: Raster and Snake stitching, related to Step 7.

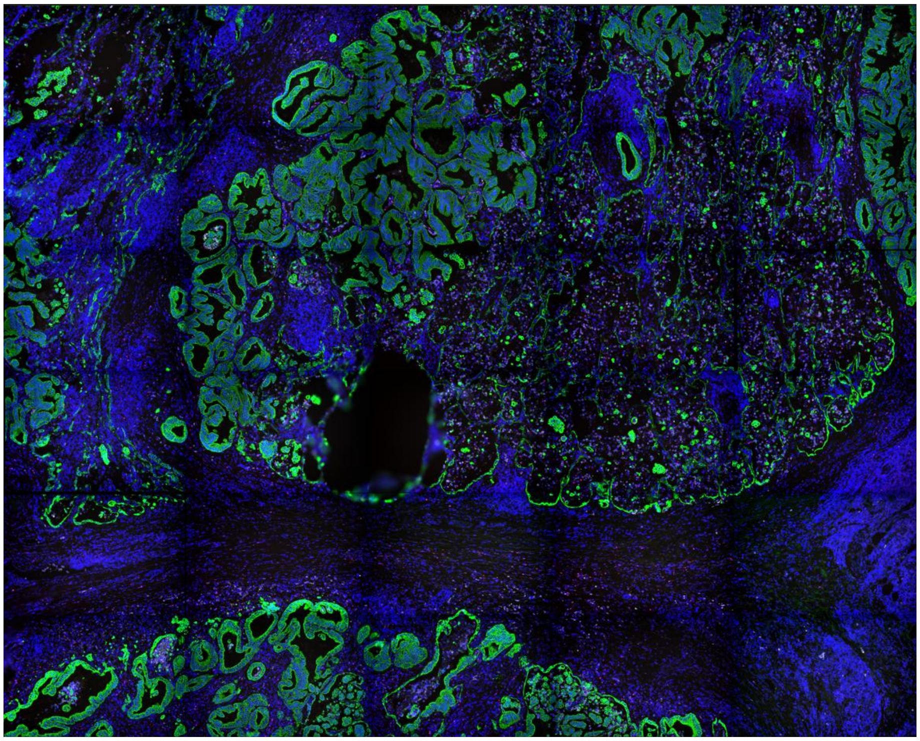

Figure S2: Stitched image for NSCLC dataset, related to Step 7.

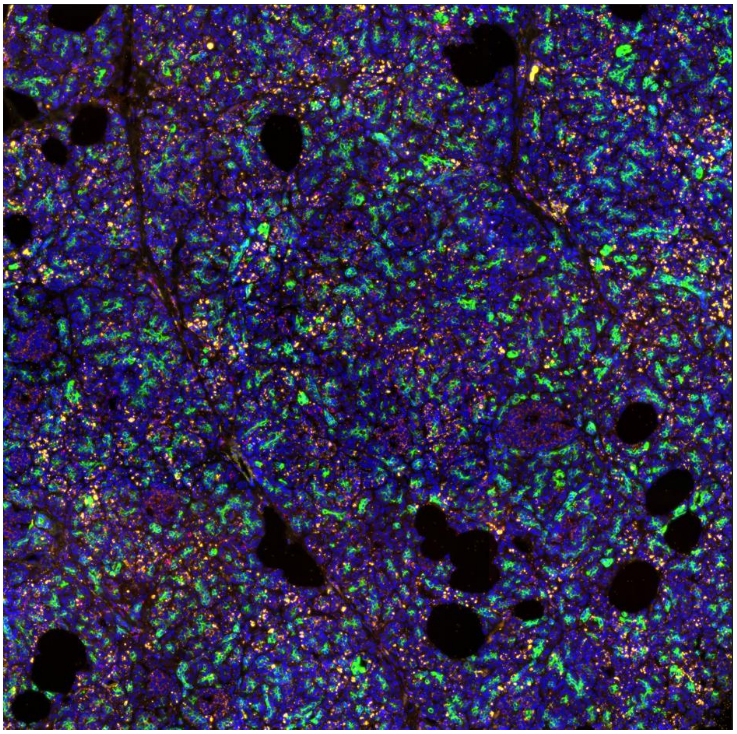

Figure S3: Stitched image for the Pancreas dataset, related to Step 7.
